# Implications of Concurrent *IDH1* and *IDH2* Mutations on Survival in Glioma—A Case Report and Systematic Review

**DOI:** 10.3390/cimb44100348

**Published:** 2022-10-21

**Authors:** Alexander Yuile, Laveniya Satgunaseelan, Joe Wei, Marina Kastelan, Michael F. Back, Maggie Lee, Heng Wei, Michael E. Buckland, Adrian Lee, Helen R. Wheeler

**Affiliations:** 1 Department of Medical Oncology, Royal North Shore Hospital, Reserve Road, St Leonards, Sydney, NSW 2065, Australia; 2 Sydney Medical School, Faculty of Medicine and Health Sciences, The University of Sydney, Sydney, NSW 2000, Australia; 3 The Brain Cancer Group, North Shore Private Hospital, Westbourne Street, St Leonards, Sydney, NSW 2065, Australia; 4 Department of Neuropathology, Royal Prince Alfred Hospital, Missenden Road, Camperdown, NSW 2050, Australia

**Keywords:** *IDH1*, *IDH2*, glioma, astrocytoma, next generation sequencing

## Abstract

Both *IDH1* (isocitrate dehydrogenase 1) and *IDH2* (isocitrate dehydrogenase 2) mutations play a vital role in the development of gliomas through disruption of normal cellular metabolic processes. Here we describe a case of a patient with an IDH-mutant astrocytoma, in which both *IDH1* and *IDH2* mutations were detected within the same tumour. The patient remains disease-free, nine and a half years after her initial diagnosis. Interrogation of cancer genomic databases and a systematic review was undertaken, demonstrating the rarity of the co-occurrence of *IDH1* and *IDH2* mutations in a variety of cancer types, and in glioma specifically. Due to the favourable outcome observed in this patient, the potential effect of concurrent *IDH1* and *IDH2* mutations on survival was also investigated.

## 1. Introduction

In 2016, the World Health Organisation (WHO) adopted isocitrate dehydrogenase 1 *(IDH1)* and isocitrate dehydrogenase 2 (*IDH2*) single nucleotide variants (SNVs) into its classification of gliomas [1]. Both genes encode IDH1 and IDH2 respectively, enzymes vital for the cellular metabolism [2]. IDH-mutant gliomas account for a larger proportion of lower grade gliomas (80% of Central Nervous System (CNS) WHO grade 2 to 3 versus 5% of CNS WHO grade 4 gliomas) as compared to IDH-wildtype gliomas. When compared to IDH-wild type WHO Grade 4 gliomas (now designated glioblastomas), IDH-mutant WHO Grade 4 astrocytomas affect patients at a younger age at diagnosis (30–40 years vs. greater than 50 years) and have longer overall survival (31 vs. 13 months). IDH- mutant WHO grade 3 astrocytomas have an even more favourable prognosis of 65 months [3]. Current diagnostic protocols now strongly recommend IDH mutational testing on all diffuse gliomas, due to its role in prognostic substratification [4]. *IDH1/2* mutational status is therefore an important molecular distinction in adult gliomas due to its prognostic value and potential as a drug target [3,5]. Since its initial discovery in gliomas [6,7], *IDH1* and *IDH2* mutations have been described in a variety of other cancers including acute myeloid leukaemia, thyroid carcinomas, cartilaginous tumours such as enchondroma and chondrosarcoma, and cholangiocarcinomas. In glioma, the vast majority of mutations are found in *IDH1* (90%) [3,8]. Whilst the most common *IDH1* mutation in gliomas result in substitute of histidine for arginine, (*IDH1* R132H), approximately 10% of mutations are at position 132, but may result in other amino acid substitution [9]. While *IDH2* mutations are common in haematological malignancies, they are relatively uncommon in gliomas, although they are also affected at an arginine residue [10].

**Figure 1:**
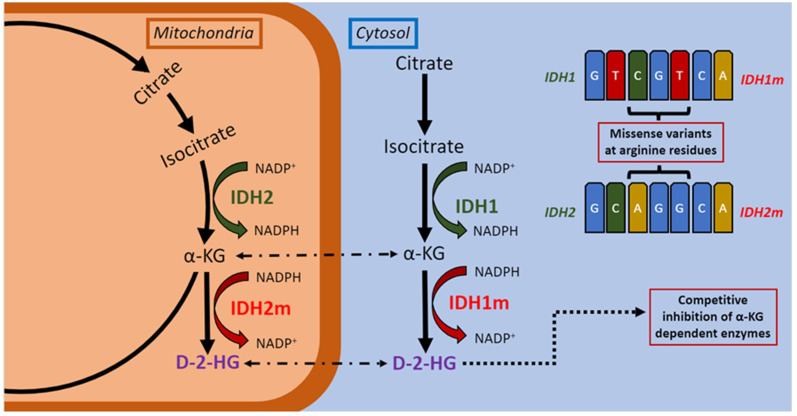
Function of wildtype IDH1/2 enzymes by intracellular compartment, with induction of oncometabolite d-2-HG by mutant IDH1/2 enzymes demonstrated.

Somatic heterozygous mutations in *IDH1* and *IDH2* correspond to changes at these substrate-binding arginine residues, located in the functional domain of the IDH enzymes (arginine 132 and arginine 172 respectively) [11,12]. The enzymatic activity of IDH1 and IDH2 catalyse the conversion of isocitrate to alpha-ketoglutarate (α-KG) in an NADP+/Mg+ dependent fashion, also yielding NADPH [13,14]. The wildtype IDH1 enzyme (encoded for by *IDH1*) is responsible for this activity in the cytosol and peroxisomes, whereas the wildtype IDH2 enzyme (encoded for by *IDH2*) governs this reaction in the mitochondria [13,14]. When either the IDH1 or IDH2 enzyme is mutant, the conversion of isocitrate to α-KG is abrogated. Instead, catalyzation of α-KG into 2-hyroxyglutarate (d-2-HG) by the mutant IDH enzyme occurs, with consumption of NADPH [3,12,14,15] (Figure 1). d-2-HG has been characterised in the literature as an oncometabolite, with its effects mediated through increased reactive oxygen species (ROS) and competitive inhibition of with α-KG-dependent enzymes [15,16]. Its effects include interference with DNA methylation resulting in a hypermethylated phenotype [17], histone methylation via histone lysine demethylases [15] and aberrant expression of hypoxia-inducible transcription factors (HIF) in response to hypoxic tumoural conditions [15,18]. Therefore, although initially suspected to have tumour suppressive function [14], *IDH1* and *IDH2* are both oncogenes, encoding oncogenic enzymes: the mutant enzymes catalyse neomorphic reactions with the resultant d-2-HG product [12].

In IDH-mutant gliomas, *IDH1* and *IDH2* mutations have typically shown mutual exclusivity [19]. Here, we describe a case of a concurrent *IDH1* and *IDH2* mutations in a patient with a CNS WHO grade 3 astrocytoma, with prolonged time to recurrence. Written informed consent was obtained from the patient and appropriately documented.

## 2. Materials and Methods

A combined survey of publicly available cancer genomic datasets and comprehensive literature search was performed. All procedures were according to the Preferred Reporting Items for Systematic Reviews and Meta Analysis (PRISMA) guidelines [20].

### 2.1. Genomic Database Interrogation

cBioPortal, a compendium of multiple genomic datasets [21], was used to interrogate a large cohort of diffuse gliomas (TCGA PanCancer Atlas—Brain Lower Grade Glioma dataset; TCGA merged cohort of LGG and GBM, 2016; UCSF Low Grade Glioma 2014 dataset; GLASS consortium diffuse glioma dataset, 2019; MSK glioma dataset, 2019). Samples with SNVs or copy number alterations in *IDH1* and/or *IDH2* were included.

### 2.2. Data Sources and Search Strategy

**Figure 2:**
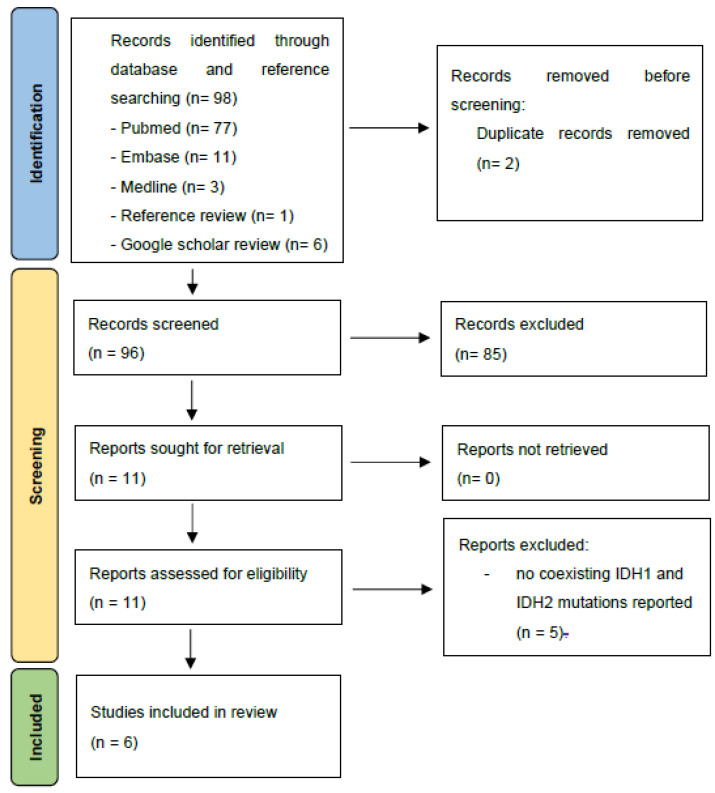
Systematic literature review strategy—PRISMA flowchart of record inclusion [20].

A systematic literature search was conducted to identify all published human data, with no language, study type or date of publication restrictions (see Figure 2). The following electronic databases were accessed:

- Pubmed (from 1966)
- Ovid MEDLINE (from 1950)
- Ovid Embase (from 1974)

Other resources searched include Google Scholar and the reference lists of retrieved articles from the database search.

### 2.3. Selection Criteria and Process

Two review authors (J.W., A.Y.) independently assessed abstracts and (in cases of uncertainty) full-text articles. Any disagreements were resolved by consensus with a third review author (H.W.). Articles in Mandarin were accepted and translated by author, JW. Eligibility criteria required articles to have identified at least one tumour with coexisting *IDH1* and *IDH2* mutations, occurring in a malignancy (pre-malignant entities such as myeloproliferative disorders were excluded).

### 2.4. Data Extraction

The following outcomes were extracted and presented for each tumour type: frequency of co-mutations, frequency of types of co-mutations, other associated genetic alterations, clinical outcomes and method of mutation assessment. If any of these findings were not stated in the article, authors were contacted to clarify these details.

## 3. Results

### 3.1. Case Presentation

A 37-year-old female presented with haemorrhage into a left frontal opercular mass in August 2012. This was managed with debulking and haematoma evacuation. She underwent further surgical debulking in November 2012. There was residual non-enhancing disease medial to surgical cavity on MRI and confirmed on Fluoroethyl-L-tyrosine Positron emission tomography (FET PET). At this time, the histopathological findings were reported as ‘consistent with a grade 3 oligoastrocytoma’. IDH-mutant status was ascertained by immunohistochemistry, whereby the canonical *IDH1* R132H mutation was identified. No 1p/19q codeletion was identified on fluorescent in situ hybridisation (FISH) testing. Using the current WHO Classification of Tumours of the Central Nervous System [22], the tumour would now be classified as an astrocytoma, IDH-mutant, CNS WHO Grade 3 [22]. The patient had no other comorbidities and no concerning family history.

The patient was managed with PET guided Intensity Modulated Radiation Therapy (IMRT) integrated boost 59.4/54Gy in 33 fractions, completed in May 2013. This was followed by 6 months of sequential temozolomide, day 1–5 every 4 weeks. The treatment was completed without complication. The patient was followed with serial MRI scans, initially every 3 months, stretching to annual reviews. The response in the tumour was reflected by the reduction in volume of the MRI T2 FLAIR weighted sequence abnormality at three months after IMRT. The patient remains well and has reached 10 years from diagnosis without evidence of recurrence.

As part of a retrospective project, DNA extracted from formalin fixed paraffin embedded tissue (FFPE) from the patient’s second tumour operation was submitted for molecular profiling with a clinically validated targeted next generation sequencing (NGS) panel (ThermoFisher, Waltham, MA, USA). NGS testing revealed concurrent pathogenic single nucleotide variants (SNVs) in *IDH1* p.Arg132His (variant allele frequency (VAF) = 42%) and *IDH2* p.Arg172Gly (VAF = 33%). Both *IDH1* and *IDH2* SNVs were confirmed via an orthogonal method, that is, clinically validated pyrosequencing assays. Also detected were a *TP53* p.Glu258Lys variant (VAF = 87%), heterozygous loss of *CDKN2A/B*, and *MYC* copy number gain. The *MGMT* promoter region was methylated as determined by pyrosequencing analysis used in a clinical diagnostic workflow. Germline testing of the patient’s blood demonstrated no familial cancer associated gene variants.

### 3.2. Genomic Database Interrogation Did Not Yield a Co-occurring IDH1 and IDH2 Mutation in Glioma Datasets

3236 samples were identified from 2847 patients. While *IDH2* copy number alterations were seen to co-occur with *IDH1* SNVs, no concurrent *IDH1* and *IDH2* SNVs were identified in this large, combined dataset.

### 3.3. Systematic Review of the Literature Shows Rare Co-occurring IDH1 and IDH2 SNVs in Cancer

We identified six publications, with one conference abstract [23] in addition (Figure 2); four publications reported co-occurring *IDH1/2* mutations in acute myeloid leukaemia (AML) [22,23,24,25], 1 in chondrosarcoma [26] and 1 in glioma [27]. Although survival data was not reported in any cases for the concurrent *IDH1* and *IDH2* mutant cases specifically, one author provided further survival information on direct contact [22] (see Table 1).

#### 3.3.1. Acute Myeloid Leukaemia

AML is the most commonly described malignancy harboring concurrent *IDH1* and *IDH2* mutations, with a frequency of 0.5% to 5.7% of all IDH-mutated AML cases [22,23,24,25]. All except one study [23] used NGS for mutation assessment in AML. In most cases, one mutation had a higher allele frequency [22,23,24,25]. Platt et al. looked for associated mutations in AML with dual *IDH1/2* mutations [22]. Of the three cases with dual *IDH1/2* mutations found (*IDH1* R132H with *IDH2* R140G, *IDH1* R132H with *IDH2* R140T and *IDH1* R132C with *IDH2* R140G), one patient had *FLT3* and *CEBPA* mutations, one had *NPM1* mutation, and one was wildtype for *FLT3*, *CEBPA* and *NPM1*. Similarly, Petrova et al. reported one case of co-occurring *IDH1* R132H and *IDH2* R140G with a co-occurring *NMP1* mutation [24]. *FLT3*, *CEPBA* and *NPM1* are commonly mutated genes in leukaemia [29].

Adequate survival data reflecting *IDH1/2* co-mutation outcomes was not reported in any of the publications. However, on personal correspondence with Platt et al., it was stated that there was no change in prognosis in patients with concurrent *IDH1/2* mutation, advising that one patient remains alive and well 24 months post allogeneic transplant, one patient surviving for 14 months post diagnosis and another at three months post diagnosis (V Nardi 2022, personal communication, June). Nayak et al. confirmed that further data was unavailable (A Nayak 2022, personal communication, August).

#### 3.3.2. Chondrosarcoma

In a study by Lugowska et al., the frequency of concurrent *IDH1* and *IDH2* mutation in chondrosarcoma was 3.8% (n = 3/80) [26]. NGS was used for mutation assessment and the combination of *IDH1* R132 and *IDH2* R172 mutations was present in 2 cases and the combination of *IDH1* R132 and *IDH2* R140 was present in one case (amino acid substitution not reported). The associated mutations found in all 3 dual *IDH1/2* mutated cases were in *TP53*, *EGFR*, *APC*, *ATM*, and *PIK3CA*. Survival data was not reported specifically for the dual *IDH1/2* mutations [26].

#### 3.3.3. Glioma

Hartmann et al. [27] provide the only published report describing concurrent *IDH1* and *IDH2* mutations in glioma. The authors screened 1010 glioma cases and found 743 cases with *IDH1/2* mutations. Of the 743 cases, 712 cases (96%) harboured the *IDH1* mutation alone, 27 cases (3.6%) harboured the *IDH2* mutation alone, and 4 cases (0.5%) harboured *IDH1* and *IDH2* mutations. Gliomas were described using the WHO 2007 classification and consisted of anaplastic astrocytoma (0.4%, n = 1/228), anaplastic oligodendroglioma (0.6%, n = 1/174) and anaplastic oligoastrocytoma (1.1%, n = 2/177) (amino acid substitutions not reported). As all four gliomas were histologically grade 3, the authors postulated that the presence of both mutations conferred a growth advantage to the constituent tumour cells. Associated mutations or survival outcomes specific to *IDH1/2* co-mutations were not reported.

## 4. Discussion

Here we describe a rare case of IDH-mutant astrocytoma with concurrent *IDH1* and *IDH2* mutations, and for the first time, detail co-occurring mutations and complete follow-up data. Hartmann et al. have described four cases of concurrent *IDH1* and *IDH2* mutations in glioma previously [27]. Interestingly, as found in our case, the tumours were also histologically WHO Grade 3. The associated molecular phenotype with concurrent *IDH1/2* mutations has not been described. Our case also demonstrated a *TP53* variant, a common finding in IDH-mutant astrocytomas [28]. Heterozygous *CDKN2A/B* deletion has not been shown to have a definitive effect on grading of IDH-mutant astrocytomas [32], and has not been previously documented in the setting of a dual *IDH1/2* mutation. *MYC* copy number gain in this setting has similarly not been described. In addition to associated mutations, the implications for survival of concurrent *IDH1/2* mutations are yet to be explored in the literature. By contrast, the presence of *MGMT* promoter methylation in this case was unsurprising [33]—*IDH1/2* variants are known to cause global hypermethylation by competitive inhibition of α-KG-dependent dioxygenases [15,30].

The individual effects of the concurrent *IDH1* and *IDH2* mutations are difficult to discern in our case. Enzymatically, IDH1 and IDH2 function in different subcellular compartments, the cytosol and mitochondria respectively [27]. It has been shown in cell culture studies that IDH1-mutant cells are reliant on the presence of IDH1-wildtype cells to provide the α-KG substrate required to produce the oncoenzyme d-2-HG in the cytosol [31]. By contrast, IDH2-mutant cells do not require the presence of IDH2-wildtype cells for local availability of α-KG in the mitochondria, and maintain d-2-HG production even in the setting of IDH2-wildtype knockdown [27]. In hypoxic states, α-KG can be converted back to isocitrate through reductive carboxylation mediated by wildtype IDH1 and IDH2, with a concomitant increase in d-2-HG [31,34,35,36,37,38]. IDH1-mutant cells display a reduced ability to induce reductive carboxylation under hypoxic circumstances, with a reliance on oxidative mitochondrial metabolism which in turn may result in decreased proliferation of tumour cells [37]. Again, much like its lack of reliance on its IDH2-wildtype counterpart, IDH2-mutant cells demonstrate an ability to sustain the reductive carboxylation pathway under hypoxia [37]. We therefore postulate that the patient’s acute presentation may have provided the hypoxic stimulus to slow tumour growth via the compromised metabolic capacity of the mutant *IDH1* allele, possibly mitigating the effects of the concurrent mutant *IDH2* allele. However, it should be noted that the timing of the *IDH1* and *IDH2* mutations in the evolution of the tumour cannot be ascertained from the information at hand. Furthermore, the metabolic interaction between mutant *IDH1* and *IDH2* alleles is yet to be elucidated in the literature.

Given the presence of a homozygous *TP53* mutation, reflective of the tumour cellularity in the sample tested (~90%), the VAFs found for each of *IDH1* and *IDH2* indicate heterozygosity of these mutations. (Our group has previously detailed a case of homozygous *IDH1* R132L mutation, with a VAF of 76% [38]. Similar to this case, no germline variants in *IDH1* or *IDH2* were detected). It follows therefore that there is likely to be substantial overlap between the tumour cells harbouring an *IDH1* and *IDH2* mutation. The possibility that both represent individual subclonal populations within the tumour, given the VAFs detected, would appear less likely. Immunohistochemical testing to assess the distribution of the mutant IDH1 and IDH2 proteins within the tumour was unfortunately unable to be performed, and is a limitation of our study of this case. 

From a clinical perspective, the testing patterns for *IDH1/2* variants have been substantially influenced by the use of immunohistochemistry for IDH1 R132H, the most common of *IDH1* and *IDH2* mutations [9]. In routine clinical diagnostic practice, the presence of positive immunohistochemical staining for IDH1 R132H within a glioma obviates the need to sequence *IDH1* or *IDH2*, as the clinically relevant mutation has been identified [4]. Therefore, it is possible that the rate of co-occurring *IDH1* and *IDH2* mutations has been underestimated. With the increased availability of NGS in clinical diagnostics, it is likely that there will be improved detection of the dual *IDH1/2* mutations in glioma, and a greater understanding of its influence on survival.

## 5. Conclusions

Concurrent *IDH1/2* mutations are rare and pose difficulty for variant curation and prognostication when encountered. To this end, our case report is the first to describe survival data in the setting of concurrent *IDH1/2* mutations. We postulate that the rate of dual *IDH1/2* mutations in gliomas is underestimated, due to the use of IDH1 R132H immunohistochemistry, and anticipate that its detection will increase with the more widespread use of NGS technologies in cancer diagnosis. While unusual, the co-ocurrence of *IDH1/2* mutations may deepen our understanding of their individual effects on glioma cell metabolism, and further studies into their clinical impact are needed.

## Figures and Tables

**Table 1:** Results of literature review.

Study	Malignancy Type	Proportion with *IDH1/2* Co-Mutation	Method of Genomic Testing	Associated Mutations	Clinical Outcomes
Meggendorfer et al. [28]	AML	7/1394 (0.5%)	NGS	Not reported	Not reported
Platt et al. [27]	AML	3/53 (5.7%)	NGS	*FLT3*	1 patient remains alive and well 24 months post allogeneic transplant, 1 patient surviving for 14 months post diagnosis and 1 patient surviving three months post diagnosis
				*CEBPA*	
				*NPM1*	
Petrova et al. [29]	AML	1/90 (1.1%)	NGS	Not reported	Not reported
Nayak et al. [26]	AML	1/33 (3.0%)	Sanger	Not reported	Not reported
			sequencing		
Lugowska et al. [30]	CS	3/80 (3.8%)	NGS	Not reported	Not reported
Hartman et al. [31]	A	1/228 (0.4%)	NGS	Not reported	Not reported
	O	1/174 (0.6%)			
	OA	2/177 (1.1%)			

AML = acute myeloid leukemia, CS = chondrosarcoma, A = astrocytoma, O = oligodendroglioma, OA = oligoastrocytoma, NGS = next generation sequencing.
